# Sub-optimal modulation of gain by the cognitive control system in young adults with early psychosis

**DOI:** 10.1038/s41398-021-01673-4

**Published:** 2021-10-27

**Authors:** Bjorn Burgher, Genevieve Whybird, Nikitas Koussis, James G. Scott, Luca Cocchi, Michael Breakspear

**Affiliations:** 1 QIMR Berghofer Medical Research Institute, Herston, QLD Australia; 2 Metro-North Mental Health Service, Brisbane, QLD Australia; 3 College of Engineering Science and Environment, College of Health and Medicine, University of Newcastle, Callaghan, NSW Australia

**Keywords:** Bipolar disorder, Schizophrenia

## Abstract

Executive dysfunctions in early psychosis (EP) are subtle but persistent, hindering recovery. We asked whether changes in the cognitive control system (CCS) disrupt the response to increased cognitive load in persons with EP. In all, 30 EP and 30 control participants undertook multimodal MRI. Computational models of structural and effective connectivity amongst regions in the CCS were informed by cortical responses to the multi-source interference task, a paradigm that selectively introduces stimulus conflict. EP participants showed greater activation of CCS regions, including the superior parietal cortex, and were disproportionately slower at resolving stimulus conflict in the task. Computational models of the effective connectivity underlying this behavioral response suggest that the normative (control) group resolved stimulus conflict through an efficient and direct modulation of gain between the visual cortex and the anterior insula (AI). In contrast, the EP group utilized an indirect path, with parallel and multi-region hops to resolve stimulus conflict at the AI. Individual differences in task performance were dependent on initial linear gain modulations in the EP group versus a single nonlinear modulation in the control group. Effective connectivity in the EP group was associated with reduced structural integration amongst those connections critical for task execution. CCS engagement during stimulus conflict is hampered in EP owing to inefficient use of higher-order network interactions, with high tonic gain impeding task-relevant (phasic) signal amplification.

## Introduction

Assertive early intervention usually yields considerable symptomatic and functional improvement in early psychosis (EP) [1]. However, cognitive deficits frequently persist, irrespective of diagnosis and treatment outcome [2, 3], and impede full recovery across social and occupational domains [4]. Such deficits are varied but frequently impact executive functions such as decision-making and cognitive flexibility. Addressing these cognitive deficits is an important component of clinical management that influences the choice of anti-psychotic medication and cognitive therapies. Characterizing the neural mechanisms of these subtle but pernicious cognitive impairments holds potential to identify new targets for patient-customized recovery programs.

The cognitive control system (CCS) is comprised of the cingulo-opercular and the fronto-parietal brain networks [5]. This system supports the maintenance and flexible implementation of higher-order plans and goals required to navigate complex social and environmental demands, such as employment and education [6]. The CCS exerts hierarchical control over lower-order sensorimotor loops [7] to fulfill roles such as integrating attention and response selection in volatile contexts [8]. Deficits in cognitive control are central to the executive dysfunction in psychotic disorders [3]. Structural and functional disturbances in individuals with psychosis consistently affect the CCS, pointing to a common diathesis that spans formal diagnoses and clinical stages. The peak age-of-onset of psychosis from late adolescence to early adulthood suggests that childhood neurodevelopment may proceed unremarkably, but a destabilization occurs in the late-maturing CCS proximal to psychosis onset [2, 7].

Studies assessing hierarchical interactions between cortical regions in EP have shown that functional changes may occur in the absence of poor performance in simple tasks [9–14]. These findings suggest that hierarchical systems only falter under high levels of task difficulty, such as that engendered by stimulus conflict. Accordingly, research suggests that although low cognitive loads can be supported by cortical systems despite deficits in connectivity, these systems cannot transition to the configuration required for high cognitive demands [15]. This is consistent with the picture of good treatment outcomes, yet persistent executive deficits.

The brain mechanism by which the CCS fails to transition from low-to-high cognitive loads in EP is unclear. To address this, we conducted a multimodal neuroimaging study using a classic stressor of the CCS, namely the multi-source interference task (MSIT) [16]. The challenging (interference) component of this task calls on the hierarchical influence of cognitive control to resolve stimulus conflict. The MSIT task elicits strong single-subject effects, with robust activation of canonical CCS regions, including anterior insula (AI), anterior cingulate cortex (ACC), dorsolateral prefrontal cortex (DLPFC), and superior parietal cortex (SPC) [17]. We hypothesized that the “cognitive stressor” of stimulus conflict would uncover network mechanisms of activation, effective connectivity, and behavior that differ between EP participants and matched controls. These were assessed using a generative framework (Dynamic Causal Modeling (DCM, [18]) and Parametric Empirical Bayes (PEB, [19]), to identify group effects and individual differences [20]. We predicted that participants in each of the two groups would respond through distinct hierarchical modes of top–down cognitive control integrating bottom–up visual input. Diffusion-Weighted Imaging (DWI) and tractography were then used to explore the relationship of these effects to structural connectivity.

## Materials and methods

### Participants

In all, 30 EP participants and 30 aged and gender-matched controls with no family history of mental health disorders were recruited from October 2017 to July 2019. The sample size for adequate power to detect differences in behavior and functional magnetic resonance imaging (fMRI) was based on similar studies examining EP populations [9–14]. All participants were 17–25 years old. EP participants had experienced a first episode of psychosis in the last 2 years and met the following eligibility criteria; (i) normal pre-morbid functioning, as rated by the Diagnostic Interview for Psychoses (DIP) [21], (ii) absence of childhood neurodevelopmental or organic brain disorder, and (iii) onset of psychosis after the age of 16. These criteria ensured EP participants were likely to have had normative neurodevelopment up until psychosis onset.

Diagnosis (EP only) and substance use (both groups) were assessed using the DIP. EP group was assessed using the Positive and Negative Symptom Scale (PANSS) [22], Social and Occupational Functioning Assessment Scale (SOFAS) [23], and Clinical Global Impression (CGI) [24] (see online Supplementary Table S1). Medication type and dose were also assessed (see online Supplementary Table S2 and S3). All participants were assessed using the Wechsler Abbreviated Scale for Intelligence [25] and the Cogstate battery for schizophrenia (www.cogstate.com). We focused on the executive functioning tasks of the Cogstate since these engaged the CCS.

All participants received written and verbal information for informed consent. All subjects under 18 years gave informed consent in addition to parental consent. The study was approved by Royal Brisbane Women’s Hospital Human Research Ethics Committee (Ref. HREC/15/QRBW/613). All procedures in this work comply with the ethical standards of the relevant national and institutional committees on human experimentation and with the Helsinki Declaration of 1975, as revised in 2008.

### MRI data acquisition and pre-processing

Data were acquired from a 3 T Siemens Prisma with a 64-channel radiofrequency head coil, including multiband (acceleration factor of 6) T2*-weighted echoplanar images (fMRI), spin echo field maps, T1-weighted MPRage images, and multi-shell diffusion-weighted echoplanar images (DWI). After pre-processing (fmriprep v20.0.6 for fMRI and MRtrix3 v3.0.2 for DWI), one control participant was excluded from analysis due to motion artifact in fMRI and DWI data. Framewise displacement (FD) (30) was similar between groups (*t* = 0.55, *df* = 57, *p* = 0.58) and all participants met minimum inclusion criteria (maximum FD < 4 mm and mean FD < 0.25 mm) (see online Supplementary Table S4 [26].

### Task-related fMRI modeling

**Fig. 1:**
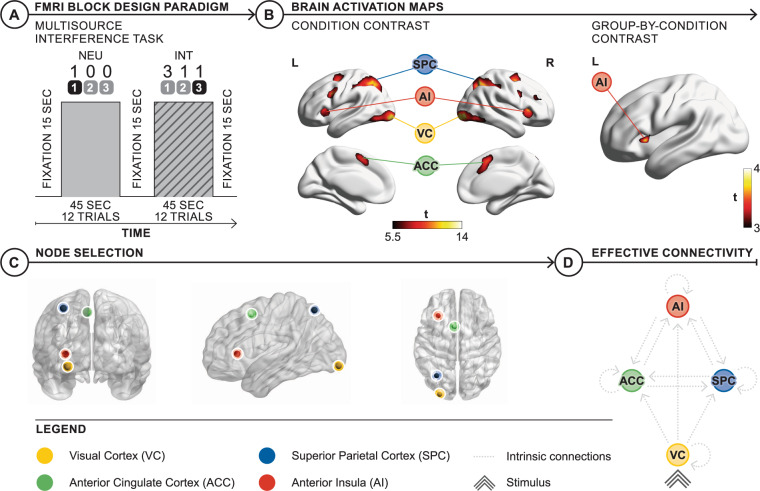
fMRI task and modeling. **A** Timing and example stimuli corresponding to neutral and interference conditions. Stimuli were presented for 2.5 s with individual trials organized in blocks of the same condition. Four blocks each of alternating neutral and interference conditions were interspersed by a fixation period. **B** Brain activation mapped to the cortical surface; canonical CCS regions showed strong and significant activation in the main effect of interference contrast (*p* < 0.001 uncorrected). The group-by-condition contrast showed increased activation in the left AI in EP participants at a permissive cluster-forming height threshold (*p* < 0.01 uncorrected). **C** Node selection for DCM model space: Time series from AI, ACC, and SPC and the visual cortex (VC) were extracted from the left hemisphere to model task-related modulations and stimulus input, respectively. **D** DCM model space common to all models: stimuli enter directly into VC, interference stimuli were able to modulate lower-order connections in a feedforward manner, with subsequent modulation of higher-order connections between ACC, SPC, and AI connected in a feedforward and feedback manner.

To investigate the effects of stimulus conflict on the CCS, fMRI data were acquired during the MSIT task (Fig. 1A). In brief, the MSIT presents stimuli organized into a group of three single digit numbers (0, 1, 2, or 3). One number is unique to the other two. Participants are instructed that this is the response target for the corresponding (first, second, or third) button press. During neutral blocks, the unique number is always in the spatial position congruent to the corresponding button and is flanked by zeros. These spatially congruent, distractor-free stimuli lead to fast responses. During interference blocks, the unique number is not in its corresponding spatial position and is flanked by numbers (1, 2, 3) that are possible responses. This requires participants to inhibit automatic responses and select the appropriate button-press based on task-relevant information, with slower reaction times reflecting spatial incongruence and distraction effects (See online Supplement for full task details). Neutral and interference trials were presented in pseudorandomized blocks of 45 s duration, interspersed by 15 s of fixation. A general linear model (GLM) of the fMRI data were performed in Statistical Parametric Mapping (SPM12, revision 7771). Regressors for trial blocks were convolved with a canonical hemodynamic response function and fitted to the blood oxygen level-dependent time series at the subject level [27]. T-contrast images were created for the main effect of task (all stimuli blocks) and interference (interference > neutral) and studied for group and interaction effects using *t* tests. Statistical inference was performed with a cluster-forming height threshold of *p* < 0.001 (uncorrected) followed by cluster-corrected inference (*p* < 0.05 FWE corrected).

**Fig. 2:**
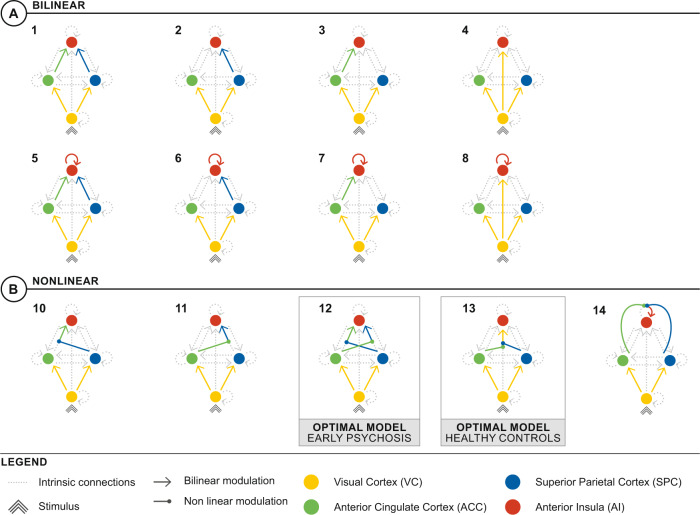
Model space. Models for the DCM were used to explore the hypothesized link between neural and behavioral task effects. Fourteen models: **A** 8 bilinear family models (exclusively linear, additive modulations with or without AI self-connection modulation), **B** 5 nonlinear family models (linear, additive modulations from VC and nonlinear, gated modulations from ACC and SPC, with or without AI self-connection modulation), and 1 null model (not shown) were included in the DCM model space as parsimonious hypotheses of modulations that explained the group-by-condition effect. The results of the BMS indicated that the optimal model for EP group was model 12 (see online Supplementary Fig. S1B) and the optimal model for the control group was model 13 (see online supplementary Fig. S1A).

We used DCM to compare hypotheses regarding effective connectivity (directed neural interactions) between the regions underlying the task effects revealed by these GLM analyses [18]. Effective connectivity during neutral and interference trials was modeled with bilinear DCMs (where neural interactions were linearly up- or downregulated by task effects) and nonlinear DCMs (where interactions are gated by activity in other regions). By incorporating gated modulations of connections between brain regions [28], nonlinear DCMs embody the manner in which cognitive control could be facilitated by hierarchical interactions across changing task contexts [5]. Canonical brain regions of the CCS that survived thresholding in the GLM analysis were incorporated into models that embodied different hypotheses about the neural substrates underlying the task (Figs. 1B, 1C and see online Supplementary Table S5A). Model space was first defined by a template that contained extrinsic (between region excitatory connections) and intrinsic connections (inhibitory self-connections) common to all models (Fig. 1D). Individual DCMs were differentiated by the way in which these template connections were modulated linearly or non-linearly by the interference condition (Fig. 2). A simple model, with no modulations, was used as a comparator.

Random effects Bayesian Model Selection (BMS) was used to identify the optimal model of effective connectivity for each of the EP and control groups [29] (see online Supplementary Fig. S1). BMS reveals how well a model explains the data of a random subject within a group (posterior model probability), how likely one model is compared to others in the comparison set (exceedance probability), and how often one model is more likely than the others accounting for the possibility that detected differences in model evidences (over subjects) are due to chance (protected exceedance probability) [30].

### Structural connectivity

We investigated associations between effective connectivity, embodied within DCMs, and estimates of structural connectivity inferred from DWI data. To derive structural connectivity, we used constrained spherical deconvolution [31] and probabilistic tractography (iFOD2) [32] to generate 100 million whole-brain streamlines between brain regions. We overlaid the subject-specific nodes generated from fMRI data on the Schaefer 400 node parcellation [33], then combined this with the streamlines to yield whole-brain structural connectivity graphs for each participant. As a summary of structural connectivity between the cortical nodes in the DCMs, we used navigability, a single measure that balances network integration and segregation [34]. Navigability sums all possible connections between two nodes in a network, returning the minimum number of hops between nodes and the weighted composite of those hops.

### Individual differences in reaction time and structural connectivity

**Fig. 3:**
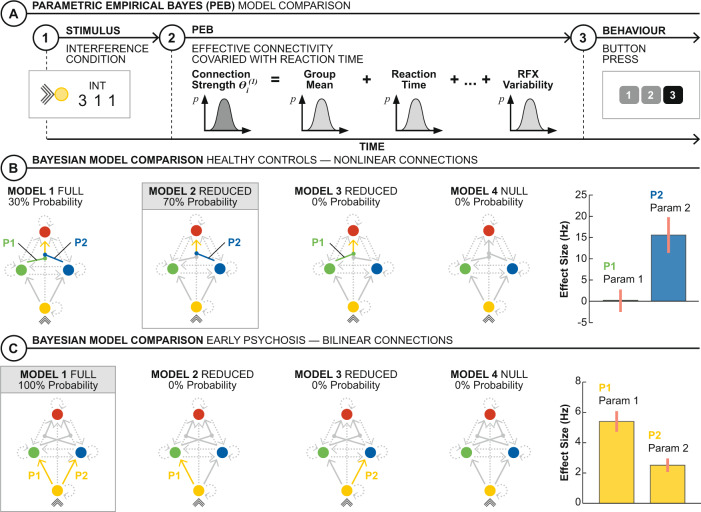
Individual differences in effective connectivity and reaction time. **A** PEB was used to model the effect of interference condition on effective connectivity on the optimal model for each group (as derived from the BMS) and its dependence on reaction time (i.e., time from stimulus onset to button press) using a random-effects analysis. **B** For controls, the gated modulation of SPC on the VC to AI connection explained most of the individual differences in reaction time with a large effect size (posterior estimate 15.6 Hz). The reduced model containing this gated modulation was the most probable model to explain individual differences in reaction time (posterior probability 70%). **C** For EP participants, the linear, additive modulations from VC to ACC and VC to SPC explained most of the reaction time variance within group with moderate effect sizes (posterior estimate 5.4 Hz and 2.5 Hz, respectively). The full model containing both these linear, additive modulations was the most probable model to explain individual differences in reaction time (posterior probability 100%).

**Fig. 4:**
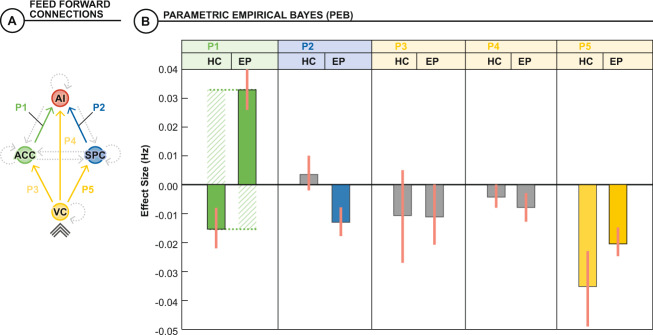
Individual differences in effective connectivity and structural connectivity. **A** PEB was used to model the effect of all trials on effective connectivity on the feedforward connections common to all models and participants (as derived from the DCM) and its covariance with navigability distance of the network’s input-output (i.e., VC to AI) using a random-effects analysis. **B** For healthy controls, higher average effective connectivity was associated with shorter network navigability distance in the ACC to AI and VC to SPC connections. For EP participants, higher connectivity was associated with longer network navigability distance in the ACC to AI connection and shorter navigability network distance in the SPC to AI and VC to SPC connections. Note that there are opposing patterns of structural-functional coupling in the ACC to AI connection between groups, which compromises the cingulo-opercular network of the CCS.

We used PEB to study the role of effective connectivity on individual differences in behavior and structural connectivity via two parameters of interest [35]: reaction time (Fig. 3) and navigability (Fig. 4), respectively. Reduced versions of the models used in each PEB (with individual connections iteratively modulated “on” or “off”) were studied to understand which parameters explained most of the variability in reaction time and navigability between subjects. The inference was performed within each group separately (i.e., using group-wise analyses). Nuisance covariates of substance misuse (both groups) and chlorpromazine equivalents [36] (EP only) were used in each PEB.

Full details of participant data, MRI data acquisition, pre-processing, task-related fMRI modeling, structural connectivity, and analyses for group effects and individual differences are provided in the supplement.

## Results

### Demographic, clinical, and cognitive assessments

The groups were matched for sex and age (see online Supplementary Table S1). Significant differences in IQ (*t* = −2.52, *df* = 58, *p* = 0.014), substance use (*χ*^2^ = 7.9, *df* = 58, *p* = 0.005) (see online Supplementary Table S6) and years of education (*t* = −3.1, *df* = 58, *p* = 0.003) were present. There were no significant differences between groups in set-shifting, as a test for cognitive control (*t* = −1.02, *df* = 58, *p* = 0.312). However, there were significant differences in the two-back (*t* = 2.44, *df* = 58, *p* = 0.018), a test of working memory. Assumptions for normal distribution and equal variances were met for two-sided *t* tests. The EP group had a mean PANSS score of 46.7 (SD = 22.6) and a mean CGI-S of 3.4 (SD = 1.6), corresponding to “mild” symptomatic levels. EP participants were generally functioning well, with a mean SOFAS score of 65.7 (SD = 15.5). Such symptom and functional profiles were in keeping with expected outpatient EP populations who are clinically stable and receiving treatment.

### Task performance

There were no significant group differences in accuracy for neutral (*t* = 0.17, *df* = 57, *p* = 0.29) or interference (*t* = 1.51, *df* = 57, *p* = 0.14) trials, nor was there a group-by-condition interaction (*f* = 1.46, *df* = 57, *p* = 0.23). However, EP participants were significantly slower in reaction times, with a minor difference in neutral trials (*t* = 2.43, *df* = 57, *p* = 0.018) and a relatively large difference in interference trials (*t* = 3.19, *df* = 57, *p* = 0.0023), yielding a significant group-by-condition interaction (*f* = 5.2, *df* = 57, *p* = 0.026) (see online Supplementary Fig. S2). Assumptions for normal distribution and equal variances were met for two-sided *t* tests and ANOVA.

### Task effects on neural activity and effective connectivity

Strong and significant effects were evident for the main effect of interference in canonical CCS regions, including the bilateral SPC, bilateral AI, bilateral ACC, and right DLPFC (all *p* < 0.001) (Fig. 1B and see online Supplementary Table S5A). The main effect of interference was also expressed bilaterally in the VC, supplementary motor area, and dorsal striatum (all *p* < 0.001). The main effect of the group revealed stronger task-evoked activations in EP participants in the bilateral SPC and the right temporal pole (all *p* < 0.001). No interaction effects were detected at the default cluster-forming height threshold of *p* < 0.001 uncorrected, despite the task inducing significant interaction effects at the behavioral level (i.e., disproportionately slower reaction times of EP participants during interference condition).

We used DCM to model the link between neural and behavioral effects. DCM is constituted by nodes—regions that embody task and group effects—and their interactions (effective connectivity), which embody the propagation and interaction of these effects. Nodes embodying stimulus, task, and group effects were informed by the significant effects discussed above. To identify a node accompanying the behavioral interaction effect, a permissive cluster-forming threshold of *p* < 0.01 uncorrected was used followed by cluster-level correction (*p* < 0.05 FWE corrected). This height threshold revealed increased activation in EP participants in the left AI for the interference > neutral t-contrast (Fig. 1C and see online Supplementary Table S5B).

To test different hypotheses about the effective connectivity underlying this neural-behavioral link, we constructed a parsimonious model space of eight bilinear models (exclusively linear, additive modulations) and five hybrid nonlinear models (linear, additive, and nonlinear, gated modulations) (Fig. 2). Although the effect of stimulus interference across groups was largely bilateral (Fig.1B and see online Supplementary Table S5A), the neural interaction effect was exclusively left-hemispheric. We, therefore, confined models to comprise left-sided nodes of the CCS, which showed interference effects, namely ACC, SPC, and AI. There was no significant group-level effect of interference in the left DLPFC, which was therefore not included in the DCMs. These models reflected various hypotheses about the modulation of AI introduced by stimulus conflict in the interference condition. In all models, VC received the stimulus input and projected feedforward connections to ACC, SPC, and AI. Effective connections to ACC and SPC were subject to bilinear modulation, incorporating the effect of stimulus conflict on these connections in the lower levels of the CCS hierarchy. Models then differed according to whether the ensuing higher level CCS connections had convergent projections to AI that were subject to linear, additive (Fig. 2A) or nonlinear, gated (Fig. 2B) modulatory effects, each embodying different mechanisms for the extra load of stimulus conflict. Bilinear and nonlinear models also differed as to whether the AI self-connection had a linear, additive (models 5–8) or nonlinear, gated (model 14) modulation. One EP and one control participant were excluded from the DCM owing to an absence of significant left AI activity. DCMs were therefore estimated from a total of 29 EP and 28 control participants.

BMS identified model 12 as the optimal model for EP participants (exceedance probability 78%, protected exceedance probability 64%) comprising bilinear modulations from VC to the more proximal ACC and SPC, with subsequent symmetrical and reciprocal gating of ACC and SPC connections to AI (see online supplementary Fig. S1B). In comparison, model 13 was optimal for controls (exceedance probability 61%, protected exceedance probability 23%), with a direct gating modulation of the more distal VC to AI connection by ACC and SPC (see online supplementary Fig S1A). In both groups, nonlinear models easily out-performed bilinear models.

### Individual differences in effective connectivity and reaction time

We next used PEB to identify which of the modulations (bilinear or nonlinear) of the optimal model within each group explains individual differences in reaction time during interference trials (Fig. 3A). For the EP participants, the low-level serial modulations from VC to ACC (posterior estimate 5.4 Hz, posterior probability >0.99) and VC to SPC (posterior estimate 2.5 Hz, posterior probability >0.99) showed a positive dependence on reaction time. The composite effect of both modulations was more probable than either, in isolation (posterior probability >99%) (Fig. 3C). Across the controls, in contrast, individual differences in the nonlinear, gated modulations better-explained reaction time differences. Specifically, the SPC-gated modulation of VC to AI showed a positive dependence on reaction time (posterior probability >0.99) with a larger effect size (posterior estimate 15.6 Hz). A reduced model containing this modulation alone was a more probable explanation of reaction time differences than models containing the other, both, and neither modulation (posterior probability 70%) (Fig. 3B). Nuisance covariates did not influence the detected individual differences in effective connectivity and reaction time.

### Individual differences in effective connectivity and structural connectivity

We used navigability as a summary measure of structural connectedness, confined to the 57 subjects included in the DCM analysis. There were no significant differences between groups in the weighted navigability amongst all CCS nodes (*t* = 1.89, *df* = 55, *p* = 0.06), nor when restricted to the connection from VC to AI, the common input–output mapping in all DCM models (*t* = 1.99, *df* = 55, *p* = 0.051). However, fewer hops between VC and AI were required in the controls than the EP participants (2.1 vs 2.4, *t* = 2.47, *df* = 55, *p* = 0.017).

The optimal DCMs for each group differed according to whether ACC and SPC-gated the VC to AI connection (control group, model 13) or not (EP group, model 12). We, therefore, chose navigability between these nodes for the analysis of individual differences between effective and structural connectivity across both task conditions, focusing on feedforward connections only (Fig. 4A). That is, using a group-wise approach, we asked whether individual differences in effective connectivity reflected individual differences in a complementary measure of the underlying structural connectivity. Among EP participants, there was a positive dependence of the effective connectivity of the ACC to AI connection and navigability distance (i.e., higher average connectivity and longer path distances; posterior effect 0.033 Hz, posterior probability >0.99). Other feedforward connections possessed a negative dependence on navigability (posterior probability >0.99), although only the SPC to AI and VC to SPC connections had effect sizes that exceeded the 90% Bayesian confidence interval (Fig. 4B). Amongst controls, in contrast, the only effects that exceeded the 90% Bayesian confidence interval were negative associations between effective connectivity of the ACC to AI (posterior effect −0.015 Hz, posterior probability >0.99) and VC to SPC (posterior effect −0.035 Hz, posterior probability >0.99) and navigability distance. The nuisance covariates did not influence the relationship between effective connectivity and navigability distance.

### Gain mapping of brain regions

**Fig. 5:**
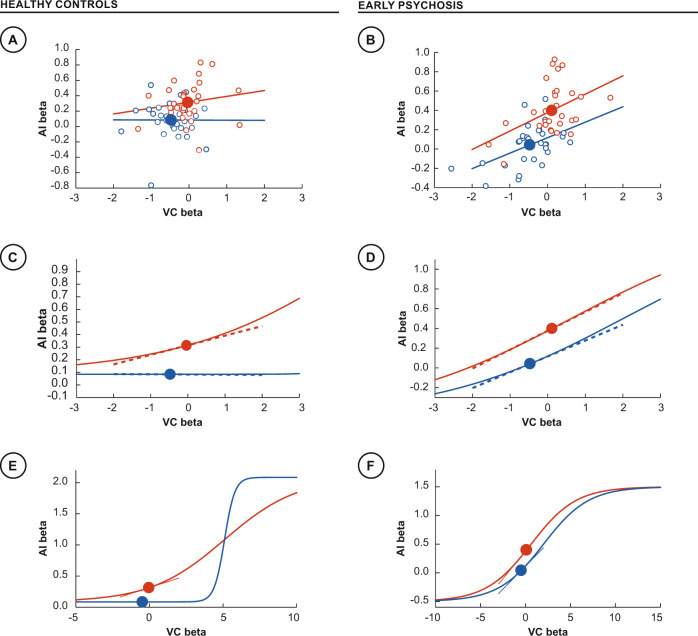
Gain control. Gain-mediated relationships between stimulus responses in the visual cortex (inputs, *x* axes) and task responses in the AI (outputs, *y* axes) using beta estimates extracted from interference > baseline and neutral > baseline t-contrasts. Top row **A**, **B** shows parameter estimates extracted from SPM contrasts in VC and AI for neutral (blue lines) and interference (red lines) conditions, with line of best fit across participants. Middle and lower rows show local (**C**, **D**) and global (**E**, **F**) sigmoid-shaped activation functions, whose local tangents yield the state-dependent input-output gain functions.

The optimal DCM models of both groups contained nonlinear, gated modulations of the AI, albeit differing in their nature. Prevailing theories of cognitive control center on up- or down-regulation in “gain” (the input-output mapping of a brain region), which is here modeled by changes in effective connectivity via DCM [28]. Gain is a nonlinear mechanism that adaptively upregulates the response of a cortical region to its inputs under higher cognitive loads (as during interference), amplifying salient stimulus features and suppressing distracting stimuli [37]. To explore this effect, we plotted the relationships between stimulus responses in VC and task-related effects in AI using subject-wise beta estimates from the interference>baseline and neutral>baseline t-contrasts (Fig. 5). Note that in our DCM models this mapping is a composite of direct and indirect connections. There is a substantial increase in this mapping from the neutral to the interference condition across the control participants (Fig. 5A). By contrast, there already exists a moderately positive mapping in the neutral task in the EP participants, with an attenuated further increase in gain in the interference task (Fig. 5B). Note that as this maps the *relative* responses of VC and AI across participants it is complementary to, but independent from the GLM analyses.

In population models of brain activity, such as those that underlie DCM for electroencephalography and magnetoencephalography, such point-wise gain mappings are drawn from an underlying sigmoid-shaped activation function [38]. To illustrate this, we fitted a canonical activation function, constrained to be tangent to the mean input-output map of each condition (Fig. 5C–F). This illustrates that a less substantial modulation is required in the EP participants to accommodate the slight increase in gain from the neutral to the interference task.

## Discussion

This study has important implications for understanding the brain mechanisms associated with cognitive impairment in EP. We observed increased activation in the SPC and a disproportionate slowing of reaction time from neutral to interference trials in EP participants. Effective connectivity in the CCS was used to identify potential neural underpinnings of this effect of stimulus conflict on reaction times. We demonstrated that reaction times for EP participants were dependent on linear, additive modulations from VC to ACC and SPC, with subsequent nonlinear, gated modulations in an additional step from these regions to the AI. In contrast, controls demonstrated simple, amplified responses by gated modulations of the connection directly linking VC to AI in a single processing step, associated with faster reaction times. Notably, the association between effective connectivity and navigability distance differed between the groups in the cingulo-opercular network (ACC to AI); an important connection that was only gated (by the SPC) in the EP group. The fact that this connection was supported by longer, less-efficient pathways in the EP participants suggests that gated modulation of this connection may have been a compensatory, less-efficient response to underlying structural changes. Additionally, the absence of structural-functional coupling in the VC to AI connection across both groups suggests that nonlinear, gated modulations were key to differences in task performance.

To further explore the importance of nonlinear, gated modulations, we mapped how network responses were amplified within each group when responding to stimulus conflict. Successful execution of interference trials requires upregulating responses to target visual stimuli, evident in the parametric effects of VC against AI across the control group (Fig. 5A). Fitting these responses to nonlinear activation functions suggests a substantial modulation of population responses in the AI under the effect of cognitive control, allowing an efficiently increased gain with only a slight increase in input (Fig. 5C, E). That is, an adaptive neural system is positioned near the lower end of the activation function, being amplified as required by the context. This supports recent multimodal (PET-fMRI) analyses of cognitive control, which suggest a low metabolic load that increases parsimoniously under high cognitive load [39]. Of note, the gain in EP participants is already positive in the neutral condition, increasing further for interference trials (Fig. 5B), indicating a tonic use of gain control across conditions. Viewed differently, these effects suggest the presence of substantial background activation in those with psychosis, consistent with ambient arousal, which then impedes the adaptive increase in gain when required. In our EP cohort, with low symptom load and high functioning, this otherwise subtle effect is only evident under the influence of stimulus conflict. This perspective converges with the DCM modeling, with the reshaping of the activation function in controls mediated by the single gated connection from ACC and SPC. In contrast, DCM analyses suggest that cognitive control in EP participants is first mediated downstream through linear, additive modulations prior to ACC and SPC. The resulting amplification is then forward propagated to AI with additional nonlinear gated modulation, but little change in the activation function.

The findings of this study highlight the importance of complex, higher-order (nonlinear) interactions across segregated brain regions when undertaking complex tasks [40]. Such an integrative function is typically mediated by highly connected brain regions within the network, which serve to support transitions in functional topology as cognitive demands increase [41]. Although we have framed this in terms of the hierarchical role of the CCS, the dynamic relationships between task-relevant regions might better be viewed as a heterarchy, in the manner in which regions within the CCS (ACC, SPC, AI) mutually reorganize their relative contributions to behavior through gain control. Gain control represents an attractive neural mechanism for cognitive control dysfunction in psychotic disorders since top–down modulation of stimulus conflict is dependent on various types of short-term plasticity [28], such as the synaptic potentiation by *N*-Methyl-d-Aspartate (NMDA) receptors. Our findings are thus in line with the disconnection hypothesis of schizophrenia, suggesting a hypofunction of NMDA receptors as the mechanism for altered short-term cortical plasticity [42].

There are several caveats to our findings. We hypothesized that the disproportionate influence of stimulus conflict on reaction times in the EP participants reflected network effects in the CCS. This hypothesis was tested using DCM, hence mandating “nodes” embodying the main effects of interference, group, and their interaction. Because there was no significant interaction effect at standard cluster-forming thresholds, we used a permissive cluster-forming threshold, hence incorporating the left AI as the “interaction node” in our DCMs. We acknowledge that this choice risks incurring type 1 error by violating the assumptions of random field theory [43, 44]. The lack of a neural interaction effect at the more-stringent cluster-forming threshold may be due to the power of our modest sample size. However, we reasoned that the disproportionate slowing of reaction times induced by stimulus conflict in the EP group should be linked to a group differential effect in the CCS. Second, we restricted our DCMs to the left hemisphere to explain our hypothesized neural-behavioral interaction. This did not permit an exploration of cognitive strategies that may use inter-hemispheric models of connectivity. Third, the EP diagnostic heterogeneity may limit syndrome-focused conclusions, but the focus on the transdiagnostic construct of cognitive control impairment provides a pragmatic avenue of inquiry that better reflects clinical reality. Fourth, differences in IQ between groups could confound differences in neural-behavioral effects. However, the comparability of the EP and control participants’ executive function may be better reflected in the similar set-shifting performance rather than the multifactorial construct of IQ. Similar group performances in set-shifting support our hypothesis that cognitive control differences may only arise in a well-functioning EP cohort once the CCS network is taxed by a sufficiently high cognitive load.

Tonic engagement of gain control—and the ensuing attenuated phasic increase—may in part explain why functional recovery is challenging for persons with EP. Educational and employment environments mimic many of the attributes of stimulus conflict, where participation in a crowded lecture or busy workplace produces high arousal and the need for focused attention. Deficits in gated modulation of gain control in people with EP may assist in identifying those who are at greater risk of functional impairment and who may require individualized management targeting cognition and functional rehabilitation. For example, when addressing cognitive deficits with cognitive remediation therapies, techniques that modify behaviors (e.g., coping skills) or environment (e.g., sensory strategies) to reduce arousal should be implemented [45]. Such adaptations would permit more scope to tune gain control adaptively. Gain control deficits may provide a novel mechanism to inform prognosis and management in people with EP.

## Supplementary information

Supplemental Material

## Data Availability

The analysis packages used in this study are freely available through the following links: fMRIPrep: https://github.com/nipreps/fmriprep, Statistical Parametric Mapping: https://www.fil.ion.ucl.ac.uk/spm/software/, Diffusion pre-processing and Tractography: https://github.com/breakspear/diffusion-pipeline and Navigability: https://github.com/caioseguin/sen_rev.
