# Supplementary material for: Sub-optimal modulation of gain by the cognitive control system in young adults with early psychosis
Source: Transl Psychiatry. 2021 Oct 27;11:549. doi: 10.1038/s41398-021-01673-4 (PMC8551269; doi:10.1038/s41398-021-01673-4)
Supplement: Supplementary file 1 — Supplemental Material [file 41398_2021_1673_MOESM1_ESM.docx]

**Sub-optimal modulation of gain by the cognitive control system in young adults with early psychosis**

***Supplementary Information***

**Contents**

Supplementary Methods and Results

Supplementary Fig. S1, S2

Supplementary Table S1, S2, S3, S4, S5A & S5B, S6

Supplementary References

**Supplementary Methods and Results**

Participants

A total of 33 early psychosis (EP) and 32 control participants were consented and recruited for the study. 2 EP and 1 control participants did not subsequently attend the experimental session; 1 EP participant was excluded on MR safety grounds (due to dental braces) and 1 control participant was excluded because of claustrophobia.

EP participants were prescribed monotherapy antipsychotic (n = 25) lithium (n = 7), antidepressants (n = 5) or a combination of antipsychotic and lithium or anti-psychotic and antidepressant (n = 4) (see online supplementary Table 2).

fMRI paradigm

To investigate the brain and behavioral effects of interference on the cognitive control system (CCS) the multisource interference task (MSIT) ^1^ was used during fMRI. The task uses visual stimuli comprising a set of 3 numbers (either “0”, “1”, “2” or “3”) that appear in center of the screen for approximately 2.5 seconds, with one number always being unique from the other two (i.e., distractors). Participants were instructed to select the identity of the unique number by button press “as quickly as possible, but do not sacrifice accuracy for speed”. 12 stimuli were contained in alternating 45 second blocks of neutral and interference condition trials, interspersed by 15 second periods of visual fixation. In total, participants undertook 4 blocks of neutral and 4 blocks of interference for a total of 96 trials. During neutral condition the unique number (“1”, “2”, “3”) was surrounded by “0”s and always located in its spatially correct position ( “1 0 0”, “ 0 2 0”, “0 0 3”). During interference condition, the unique number (“1”, “2”, “3”) was flanked by distracting numbers e.g. (e.g. “1 3 1”) to produce a flanker effect^2^. Additionally, the unique number was never in its correct spatial position (e.g., “3 1 3”), thereby producing a spatial incongruence effect. Accuracy and reaction time during trials were acquired to measure the effect of interference. All participants undertook a practice session on a laptop outside of the MRI scanner. This session involved one block of neutral and one block of interference trials. Once participants understood the task, they undertook the full task in the scanner.

MRI data acquisition

T2*-weighted echoplanar images (EPI) were acquired in a posterior-anterior (PA) phase encoding direction, consisting of 60 slices of 2.4mm thickness in interleaved order, obtained with a repetition time (TR) = 82 ms, echo time (TE) = 3.3ms, flip angle = 53 degrees, field of view (FOV) = 206mm x 206mm, in-plane resolution = 86 x 86 and acceleration factor of 6. Spin echo field maps were obtained in PA and anterior-posterior (AP) directions, containing 60 slices with a TR = 6350ms and TE = 63ms. T1-weighted (T1w) MPRage images consisted of 208 slices of 0.8mm slice thickness in interleaved order, with a TR = 2400ms, TE = 2.22 ms, flip angle = 8 degrees, FOV = 256mm x 256mm. Diffusion-weighted images were acquired in AP direction, consisting of 60 non-collinear diffusion directions at b = 3000 s/mm^2^, 20 directions at b = 1000 s/mm^2^, 10 directions at b = 500 s/mm^2^, 6 directions at b = 200 s/mm^2^, and 11 directions at b = 0 s/mm2. The diffusion weighted images contained 110 slices of 2 mm^3^ thickness, obtained with a TR = 4.1 s, TE = 84.0 ms and acceleration factor of 2. Diffusion weighted images were also captured in the PA direction with same parameters using 11 directions at b = 0s/mm^2^.

Task fMRI pre-processing

Task data were pre-processed with fmriprep (version 20.0.6) ^3^and an in house (fmripop; RRID:SCR_001362) Nipype based tool ^4^ was used to regress fMRI confounds of motion, cerebrospinal fluid (CSF) and white matter(WM)), smooth data (full half width maximum 8mm) and censor volumes from the pre-processed outputs.

As quoted verbatim from fmriprep pre-processing output:

The T1-weighted (T1w) image was corrected for intensity non-uniformity (INU) with N4BiasFieldCorrection ^5^, distributed with ANTs 2.2.0 (^6^; RRID:SCR_004757), and used as T1w-reference throughout the workflow. The T1w-reference was then skull-stripped with a Nipype implementation of the antsBrainExtraction.sh workflow (from ANTs), using OASIS30ANTs as target template. Brain tissue segmentation of cerebrospinal fluid (CSF), white-matter (WM) and gray-matter (GM) was performed on the brain-extracted T1w using fast (FSL 5.0.9, RRID:SCR_002823,(^7^). Brain surfaces were reconstructed using recon-all (FreeSurfer 6.0.1, RRID:SCR_001847,^8^), and the brain mask estimated previously was refined with a custom variation of the method to reconcile ANTs-derived and FreeSurfer-derived segmentations of the cortical gray-matter of Mindboggle (RRID:SCR_002438,^9^). Volume-based spatial normalization to one standard space (MNI152NLin6Asym) was performed through nonlinear registration with antsRegistration (ANTs 2.2.0), using brain-extracted versions of both T1w reference and the T1w template. The following templates were selected for spatial normalization: FSL’s MNI ICBM 152 non-linear 6th Generation Asymmetric Average Brain Stereotaxic Registration Model [^10^, RRID:SCR_002823; TemplateFlow ID: MNI152NLin6Asym].

For each of the 1 BOLD runs found per subject (across all tasks and sessions), the following preprocessing was performed. First, a reference volume and its skull-stripped version were generated using a custom methodology of fMRIPrep. A B0-nonuniformity map (or fieldmap) was estimated based on two (or more) echo-planar imaging (EPI) references with opposing phase-encoding directions, with 3dQwarp ^11^(AFNI 20160207). Based on the estimated susceptibility distortion, a corrected EPI (echo-planar imaging) reference was calculated for a more accurate co-registration with the anatomical reference. The BOLD reference was then co-registered to the T1w reference using bbregister (FreeSurfer) which implements boundary-based registration ^12^. Co-registration was configured with six degrees of freedom. Head-motion parameters with respect to the BOLD reference (transformation matrices, and six corresponding rotation and translation parameters) are estimated before any spatiotemporal filtering using mcflirt (FSL 5.0.9,^13^). BOLD runs were slice-time corrected using 3dTshift from AFNI 20160207 (^11^, RRID:SCR_005927). The BOLD time-series (including slice-timing correction when applied) were resampled onto their original, native space by applying a single, composite transform to correct for head-motion and susceptibility distortions. These resampled BOLD time-series will be referred to as preprocessed BOLD in original space, or just preprocessed BOLD. The BOLD time-series were resampled into several standard spaces, correspondingly generating the following spatially-normalized, preprocessed BOLD runs: MNI152NLin6Asym. First, a reference volume and its skull-stripped version were generated using a custom methodology of fMRIPrep. Several confounding time-series were calculated based on the preprocessed BOLD: framewise displacement (FD), DVARS and three region-wise global signals. FD and DVARS are calculated for each functional run, both using their implementations in Nipype ^14^. The three global signals are extracted within the CSF, the WM, and the whole-brain masks. Additionally, a set of physiological regressors were extracted to allow for component-based noise correction (CompCor,^15^). Principal components are estimated after high-pass filtering the preprocessed BOLDtime-series (using a discrete cosine filter with 128s cut-off) for the two CompCor variants: temporal (tCompCor) and anatomical (aCompCor). tCompCor components are then calculated from the top 5% variable voxels within a mask covering the subcortical regions. This subcortical mask is obtained by heavily eroding the brain mask, which ensures it does not include cortical GM regions. For aCompCor, components are calculated within the intersection of the aforementioned mask and the union of CSF and WM masks calculated in T1w space, after their projection to the native space of each functional run (using the inverse BOLD-to-T1w transformation). Components are also calculated separately within the WM and CSF masks. For each CompCor decomposition, the k components with the largest singular values are retained, such that the retained components’ time series are sufficient to explain 50 percent of variance across the nuisance mask (CSF, WM, combined, or temporal). The remaining components are dropped from consideration. The head-motion estimates calculated in the correction step were also placed within the corresponding confounds file. The confound time series derived from head motion estimates and global signals were expanded with the inclusion of temporal derivatives and quadratic terms for each ^16^. Frames that exceeded a threshold of 0.5 mm FD or 1.5 standardised DVARS were annotated as motion outliers. All resamplings can be performed with a single interpolation step by composing all the pertinent transformations (i.e. head-motion transform matrices, susceptibility distortion correction when available, and co-registrations to anatomical and output spaces). Gridded (volumetric) resamplings were performed using antsApplyTransforms (ANTs), configured with Lanczos interpolation to minimize the smoothing effects of other kernels ^17^. Non-gridded (surface) resamplings were performed using mri_vol2surf(FreeSurfer).

Many internal operations of fMRIPrep use Nilearn 0.6.2 (^18^, RRID:SCR_001362), mostly within the functional processing workflow.

Tractography, parcellation and network measures

We registered our participants using non-linear warping from ANTs ^19^ to MNI152 space to back propagate the registration of the Schaefer 400 node parcellation ^20^ to individual subject space. We selected the 400-node scale to overlay four 8mm spheres centered at the four maxima of the functional activations in the interference-neutral contrast used in the Dynamic Causal Modelling (DCM) analysis. Seeding was random across the surface of the grey matter/white matter interface and propagated using the iFOD2 algorithm ^21^. 100 million streamlines were seLected based on the following parameters: 1mm step size, maximum 45 degree angle between steps, 10/250 mm min./max. lengths, 0.1 FOD amplitude cut-off, 1000 trials per seed, terminate at grey matter/white matter interface. Streamlines are stopped once they reach the termination criterion.

The SIFT2 algorithm was performed on each tractogram ^22^, and streamlines counts were summed for each connection in the 400+4-node parcellation mentioned above, resulting in a connectome for each subject. Consistency-based thresholding of connection weights was then applied across groups to filter out spurious connections ^23^. A density threshold of 10% was chosen, keeping the top 10% most consistent. Connection length matrices were computed from the weighted fiber density to ensure log-normally distributed connection lengths and attenuation of extreme connection weights ^24^. Physical connection distance matrices were computed as the Euclidean distance between the centroids of each node in the 400+4 parcellation.

Navigation on these networks was performed on a subject level using each converted connection-length matrix and matching Euclidean distance matrix (navigation_wu.m in brain connectivity toolbox) ^25^. From this three metrics were derived; the navigation path distance between DCM node pairs the sum of connection weights along the path; and the number of binary jumps between nodes along the path.

Task-related fMRI: Paradigm, analysis and modelling

Statistical inference was performed using a cluster-forming height threshold of p < 0.001 uncorrected. However, the ensuing effects were also highly significant with the default cluster-forming threshold, although many of the clusters were confluent in extent. For example, the anterior cingulate cortex (ACC) was confluent with the bilateral supplementary motor areas (SMA). In this instance, the ACC cluster maxima was isolated from the SMA using an extremely stringent cluster-forming height threshold of p < 0.00005 FWE.

The BOLD time series for the DCM nodes were then extracted. BOLD time series in each node, for each subject, was extracted using a 5mm radius sphere mask derived from the subject level maxima of the f-contrast image with a cluster-forming height threshold of p < 0.05 uncorrected. Participants that did not have signal in the brain region of interest after this thresholding were not included in the DCM. One EP and one control participant were excluded from the DCM due to an absence of left anterior insula (AI) signal at p < 0.05 threshold. The 5mm sphere used to define the region of interest was contained within a mask of the t-contrast cluster derived from the group level maxima of the main effect of interference. However, the mask used to capture the BOLD time series for the left AI was a conjunction of the group level maxima condition contrast and a bipartite insula parcellation derived from connectivity profiles associated with anterior and posterior regions ^26^. This decision was motivated by the need to capture the AI more specifically; since previous work has suggested that the functional division of anterior and posterior insula is different in psychotic disorders compared to normative populations.

Individual differences in effective connectivity and reaction time

Parametric Empirical Bayes (PEB) was used to identify dependences between the modulation of effective connectivity by the effect interference and individual differences in reaction time in a group-wise analysis. As different group-wise optimal models were identified with Bayesian Model Selection (Fig. 3 A,B), separate PEB analyses were conducted for each group. Furthermore, since each group’s optimal models utilized both linear and nonlinear modulations, separate PEB analyses were conducted for each modulation type within each group. Alternative models were compared against the full model (i.e., each group’s optimal model). Alternative models for each PEB were reduced versions of the full optimal model by iteratively modulating connections “on” or “off”.

Neither the nonlinear PEB for EP participants (i.e., nonlinear, gated modulations covaried against reaction time) nor the bilinear PEB for controls (i.e., linear, additive modulations covaried with reaction time) reached a posterior probability of >0.99.

Individual differences in effective connectivity and navigability

PEB was then used to identify which connections modulated during main effect of task (i.e., both neutral and interference conditions) were associated with individual differences in navigability for each group. Effective connectivity in this instance was estimated from the template DCM that contained all intrinsic connections between the visual cortex (VC) and the CCS brain regions (Fig. 1A). A single alternative model of all feedforward connections (Fig. 5A) turned “off” was compared against the full model (i.e., the template DCM). For each group every parameter contributed to the model evidence with a posterior probability of >0.99.

Gain mapping of brain regions

As a post-hoc analysis we explored the effect of input-output gain control in the AI by plotting the relationships between stimulus responses in VC (input) and task-related effects in AI (output) across participants. Beta values from the GLM analyses were extracted from the t-contrast images of neutral > baseline and interference > baseline using the same region of interest masks for VC and AI used in the DCM. Beta values for VC and AI were plotted against each other according to contrast and group in point-wise fashion. Linear gain relationships were fitted to these data using ordinary least squares regression (Fig. 5A, B). Within each group, underlying activations functions of the form,

$$f\left( x \right)=\left( 1+tanh\left( \frac{x-X}{\sigma} \right) \right)+Y,$$

were the estimated, constrained to be tangent at the midpoint (means) of each of these gain functions (Fig. 5C-F). To ensure these functions could be uniquely estimated, the following constraints were enforced: (1) Within each group (EP, controls) the linear offset Y had to be equal for both conditions (neutral, interference); (2) Consistent with neurobiology, the gain functions had to be tangent to the activation curves below the point of inflexion^27^; only 1 of the 2 remaining free parameters could be varied between conditions – the left-right offset (X), or the nonlinear width parameter $\sigma$. Parameters for the control group (Fig.5C, E) were (Y=0.085, X=5.0, $\sigma$=0.7 (neutral), $\sigma$=5.0 (interference)). Parameter values for the EP group were (Y=-0.5; X=2.006 (neutral), X=0.625 (interference), $\sigma$=5.0).

**Figures**

**Figure S1: Bayesian Model Selection**


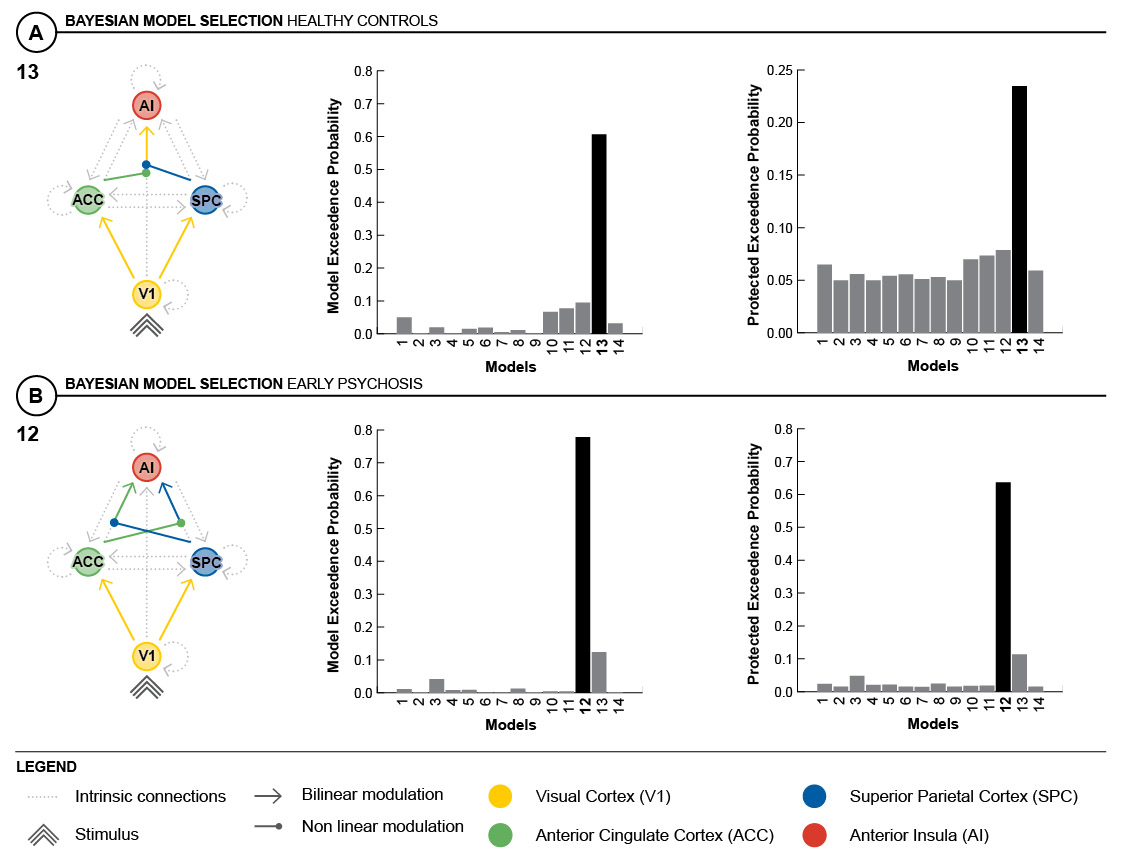


Fig. S1. Results of Bayesian Model Selection, comparing the 14 models within each group. The exceedance probability quantifies how likely one model is compared to other models, when considering the group data. The protected exceedance probability quantifies how likely the model evidence is above and beyond chance. Both groups had a singular “optimal” model containing nonlinear modulations: model 13 for EP group and model 12 for healthy control group. (A)The healthy control model directly conveyed stimulus conflict from VC to AI through higher order, nonlinear, gated modulations. (B) EP group used lower level, linear, additive modulations to convey stimulus conflict to the CCS regions, before using additional higher order nonlinear, gated modulations to convey stimulus conflict to AI.

**Figure S2: Group-by-condition interaction in task performance – reaction time**

Fig. S2. Difference in mean reaction time between interference and neutral conditions plotted for each participant, with participants categorized by group.

**Supplementary Tables**

**Table S1 Clinical and Demographic Detail of Participants**

|  | **Healthy Control (N = 30)** | | **Early Psychosis (N = 30)** | |
| --- | --- | --- | --- | --- |
|  | Mean | SD | Mean | SD |
| **Characteristics** | | | | |
| Age (years) | 21.8 | 2.2 | 21.3 | 1.9 |
| Years of Education^1^ | 15.1 | 1.7 | 13.7 | 1.8 |
| WASI-II IQ estimate^1^ | 118.2 | 10.1 | 111.1 | 11.3 |
| Chlorpromazine eq. (mg) |  |  | 178 | 134 |
| PANSS |  |  | 46.7 | 22.6 |
| CGI-Severity |  |  | 3.4 | 1.6 |
| SOFAS |  |  | 65.7 | 15.5 |
|  |  |  |  |  |
|  | N | % | N | % |
| Male | 16 | 53.3 | 17 | 56.7 |
| Substance Use^1^ | 6 | 21.0 | 18 | 60.0 |
| Antipsychotic naïve |  |  | 5 | 16.6 |
| Antidepressant |  |  | 5 | 16.6 |
| Lithium |  |  | 7 | 23.3 |
| Combination^2^ |  |  | 4 | 13.3 |
| Schizophrenia spectrum^3^ |  |  | 18 | 60.0 |
| Bipolar spectrum^3^ |  |  | 12 | 40.0 |
| Single episode of psychosis |  |  | 11 | 36.7 |
| Relapse of psychosis |  |  | 19 | 63.3 |

Table S2. Participants were matched for age and gender (i.e., no significant differences between groups p > 0.05). “High Frequency” substance use was defined as any use of alcohol, tobacco or narcotic of a frequency greater than 1-2 times per week i.e., a score of 3 or higher for any type of substance use, according to the Diagnostic Interview for Psychoses.

^1^ Early psychosis group had significantly lower years of Education (t=-3.1, p=0.03, df=57), lower estimate of IQ (t=-2.52, p=0.01 df=57) and higher past year substance use (χ^2^=7.9, p=0.01, N=57).

^2^ Combination medications included antipsychotic and antidepressant or antipsychotic and lithium. See Table S1.

^3^ As per ICD-10 World Health Organization^28^, schizophrenia spectrum disorders in this study included schizophrenia, schizoaffective disorder, acute and transient psychotic disorder, other primary psychotic disorders, unspecified primary psychotic disorders. Bipolar spectrum disorders in this study include bipolar affective disorder with a history of mania with psychotic symptoms.

**Table S2: Medication Types**

| **Antipsychotic Type** | **Oral** | **Depot** |
| --- | --- | --- |
| Risperidone | 6 |  |
| Paliperidone |  | 4 |
| Olanzapine | 2 |  |
| Quetiapine | 1 |  |
| Clozapine | 1 |  |
| Amisulpride | 1 |  |
| Aripiprazole | 3 | 6 |
| Brexpiprazole | 1 |  |
| Lurasidone | 1 |  |
|  |  |  |
| **Antidepressant Type** | **Oral** |  |
| Sertraline | 3 |  |
| Fluoxetine | 1 |  |
| Citalopram | 1 |  |
|  |  |  |
| **Mood stabilizer Type** | **Oral** |  |
| Lithium | 7 |  |
| **Combination Type** | **Antipsychotic, Lithium** | **Antipsychotic, Antidepressant** |
|  | 2 | 2 |
|  |  |  |

**Table S3: Antipsychotic dose**

| **Subject** | **Chlorpromazine equivalents in milligrams (CPZ)** |
| --- | --- |
| sub-01 | 133 |
| sub-02 | 200 |
| sub-03 | 200 |
| sub-04 | 300 |
| sub-05 | 100 |
| sub-06 | 0 |
| sub-07 | 100 |
| sub-08 | 0 |
| sub-09 | 133 |
| sub-10 | 133 |
| sub-11 | 0 |
| sub-12 | 100 |
| sub-13 | 400 |
| sub-14 | 0 |
| sub-15 | 200 |
| sub-16 | 200 |
| sub-17 | 266 |
| sub-18 | 200 |
| sub-19 | 200 |
| sub-20 | 333 |
| sub-21 | 266 |
| sub-22 | 300 |
| sub-23 | 266 |
| sub-24 | 200 |
| sub-25 | 600 |
| sub-26 | 200 |
| sub-27 | 200 |
| sub-28 | 0 |
| sub-29 | 133 |
| sub-30 | 0 |

**Table S4: fMRI head motion**

| **Subject** | **Maximum Framewise Displacement (mm)** | **Mean Framewise Development (mm)** |
| --- | --- | --- |
| sub-01 | 0.419 | 0.059 |
| sub-02 | 0.675 | 0.103 |
| sub-03 | 1.643 | 0.101 |
| sub-04 | 0.217 | 0.080 |
| sub-05 | 0.905 | 0.209 |
| sub-06 | 0.279 | 0.104 |
| sub-07 | 0.424 | 0.139 |
| sub-08 | 0.65 | 0.137 |
| sub-09 | 0.769 | 0.221 |
| sub-10 | 0.199 | 0.069 |
| sub-11 | 0.235 | 0.091 |
| sub-12^1^ | 4.193 | 0.191 |
| sub-13 | 0.488 | 0.174 |
| sub-14 | 2.414 | 0.187 |
| sub-15 | 0.414 | 0.141 |
| sub-16 | 2.625 | 0.207 |
| sub-17 | 1.917 | 0.219 |
| sub-18 | 0.552 | 0.162 |
| sub-19 | 0.351 | 0.097 |
| sub-20 | 0.396 | 0.111 |
| sub-21 | 0.828 | 0.145 |
| sub-22 | 0.568 | 0.138 |
| sub-23 | 0.387 | 0.097 |
| sub-24 | 0.471 | 0.177 |
| sub-25 | 0.545 | 0.109 |
| sub-26 | 0.724 | 0.104 |
| sub-27 | 0.952 | 0.240 |
| sub-28 | 0.408 | 0.085 |
| sub-29 | 0.222 | 0.096 |
| sub-30 | 0.424 | 0.196 |
| sub-31 | 0.641 | 0.187 |
| sub-32 | 0.673 | 0.167 |
| sub-33 | 1.201 | 0.229 |
| sub-34 | 0.58 | 0.209 |
| sub-35 | 0.382 | 0.159 |
| sub-36 | 0.44 | 0.140 |
| sub-37 | 0.405 | 0.159 |
| sub-38 | 0.212 | 0.084 |
| sub-39 | 1.569 | 0.143 |
| sub-40 | 0.283 | 0.126 |
| sub-41 | 0.22 | 0.094 |
| sub-42 | 0.523 | 0.158 |
| sub-43 | 0.99 | 0.146 |
| sub-44 | 0.265 | 0.105 |
| sub-45 | 0.33 | 0.086 |
| sub-46 | 1.336 | 0.203 |
| sub-47 | 0.247 | 0.113 |
| sub-48 | 0.318 | 0.111 |
| sub-49 | 0.408 | 0.103 |
| sub-50 | 0.473 | 0.082 |
| sub-51 | 0.722 | 0.110 |
| sub-52 | 0.213 | 0.085 |
| sub-53 | 0.323 | 0.057 |
| sub-54 | 0.506 | 0.100 |
| sub-55 | 0.548 | 0.139 |
| sub-56 | 0.322 | 0.166 |
| sub-57 | 2.996 | 0.249 |
| sub-58 | 1.347 | 0.191 |
| sub-59 | 0.329 | 0.080 |

Table S4. 1 Movement spike occurred at 400 seconds during fixation period at end of task, and therefore was not regressed in GLM or incorporated in DCM. Subject was therefore included in analysis.

Sub-01 to Sub-30: Early Psychosis.

Sub-31 to Sub-59: Healthy Control.

**Table S5A Main effects on neural activity at cluster-forming height threshold p < 0.001 uncorrected**

| **Cluster region** | **Peak co-ordinates**  **(x,y,z)** | **Peak Level t** | **Peak level z** | **Peak level P**  **(FWE corrected)** | **Cluster-level P**  **(FWE corrected)** | **Cluster size**  **(voxels)** |
| --- | --- | --- | --- | --- | --- | --- |
| **Main Effect of Task t contrast** | | | | | | |
| Right Visual Cortex | 8 -76 -19 | 24.24 | Inf | 0.00 | 0.00 | 1922 |
| Right Superior Parietal Cortex | 49 -33 46 | 13.88 | Inf | 0.00 | 0.00 | 2294 |
| Left Cerebellum | -30 -71 -50 | 9.51 | 7.35 | 0.00 | 0.00 | 170 |
| Left Visual Cortex | -13 -97 0 | 7.55 | 6.27 | 0.00 | 0.00 | 129 |
| **Main Effect of Interference t contrast** | | | | | | |
| Right Visual Cortex | 27 -97 -10 | 13.06 | Inf | 0.00 | 0.00 | 2677 |
| Left Visual Cortex^3^ | -35 -92 -12 | 13.05 | Inf | 0.00 | 0.00 | 1373 |
| Right Superior Parietal Cortex | 30 -66 48 | 13.04 | Inf | 0.00 | 0.00 | 1960 |
| Left Superior Parietal Cortex^1^ | -28 -64 53 | 11.92 | Inf | 0.00 | 0.00 | 2306 |
| Left Supplementary Motor Area^1,2^ | -47 6 34 | 11.33 | Inf | 0.00 | 0.00 | 3101 |
| Right Anterior Insula | 32 22 5 | 10.95 | Inf | 0.00 | 0.00 | 586 |
| Left Anterior Insula^1^ | -30 22 5 | 10.34 | 7.75 | 0.00 | 0.00 | 414 |
| Right Supplementary Motor Area | 47 6 31 | 8.21 | 6.66 | 0.00 | 0.00 | 253 |
| Right Dorsal Striatum | 18 -23 22 | 8.07 | 6.58 | 0.00 | 0.00 | 255 |
| Left Dorsal Striatum | -16 -18 24 | 7.44 | 6.21 | 0.00 | 0.00 | 145 |
| Right Dorsolateral Prefrontal Cortex | 47 32 26 | 7.26 | 6.10 | 0.00 | 0.00 | 314 |
| **Main Effect of Group – Early Psychosis > Healthy Controls** | | | | | | |
| Right Superior Parietal Cortex | 44 -37 60 | 4.94 | 4.49 | 0.104 | 0.00 | 898 |
| Right Temporal Pole | 37 13 -41 | 4.93 | 4.48 | 0.106 | 0.03 | 570 |
| Left Superior Parietal Cortex | -37 -42 53 | 4.30 | 3.98 | 0.504 | 0.03 | 562 |

**Table S5B Interaction effects at cluster-forming threshold of p < 0.01 uncorrected**

| **Cluster region** | **Peak co-ordinates**  **(x,y,z)** | **Peak Level t** | **Peak level z** | **Peak level P**  **(FWE corrected)** | **Cluster-level P**  **(FWE corrected)** | **Cluster size**  **(voxels)** |
| --- | --- | --- | --- | --- | --- | --- |
| **Early Psychosis > Healthy Control in the Interference > Neutral contrast** | | | | | | |
| Left Anterior Insula^4^ | -37 15 0 | 3.89 | 3.65 | 0.89 | 0.01 | 641 |

Table S5A. ^1^ Canonical CCS regions in the left hemisphere used for DCM.

^2^ Anterior Cingulate Cortex was confluent with the Supplementary Motor Areas bilaterally and required a cluster-forming height threshold of p< 0.00005 to identify as a separate cluster.

^3^ Left Visual Cortex was used as the stimulus input for DCM due to its strong effect in main effect of conflict (interference > neutral) contrast.

Table S5B. ^4^ Interaction effects were not detected at the stringent cluster-forming height threshold of p < 0.001 uncorrected. Use of the p < 0.01 uncorrected threshold was performed to detect weaker interaction effects, which was found in the Left Anterior Insula. This motivated the DCM hypothesis regarding the interaction effects in task behavior, at the risk of incurring type 1 error.

**Table S6: Substance use frequency in the past 12 months**

| Subject | Alcohol | Illicit | Tobacco | Any High Frequency Substance Use (Alcohol, illicit or tobacco) |
| --- | --- | --- | --- | --- |
| sub-01 | 1 | 1 | 1 | 1 |
| sub-02 | 0 | 0 | 0 | 0 |
| sub-03 | 0 | 1 | 0 | 1 |
| sub-04 | 1 | 1 | 1 | 1 |
| sub-05 | 0 | 0 | 0 | 0 |
| sub-06 | 0 | 0 | 0 | 0 |
| sub-07 | 1 | 0 | 0 | 1 |
| sub-08 | 1 | 1 | 0 | 1 |
| sub-09 | 1 | 1 | 0 | 1 |
| sub-10 | 0 | 0 | 0 | 0 |
| sub-11 | 0 | 0 | 0 | 0 |
| sub-12 | 0 | 1 | 1 | 1 |
| sub-13 | 0 | 1 | 1 | 1 |
| sub-14 | 0 | 0 | 0 | 0 |
| sub-15 | 0 | 0 | 0 | 0 |
| sub-16 | 0 | 1 | 1 | 1 |
| sub-17 | 0 | 1 | 0 | 1 |
| sub-18 | 0 | 1 | 0 | 1 |
| sub-19 | 0 | 1 | 1 | 1 |
| sub-20 | 0 | 0 | 0 | 0 |
| sub-21 | 0 | 0 | 0 | 0 |
| sub-22 | 1 | 1 | 1 | 1 |
| sub-23 | 1 | 1 | 0 | 1 |
| sub-24 | 0 | 0 | 0 | 0 |
| sub-25 | 0 | 1 | 0 | 1 |
| sub-26 | 1 | 0 | 0 | 1 |
| sub-27 | 1 | 0 | 1 | 1 |
| sub-28 | 0 | 0 | 0 | 0 |
| sub-29 | 0 | 0 | 0 | 0 |
| sub-30 | 0 | 1 | 0 | 1 |
| sub-31 | 0 | 0 | 0 | 0 |
| sub-32 | 0 | 0 | 0 | 0 |
| sub-33 | 0 | 0 | 0 | 0 |
| sub-34 | 0 | 0 | 0 | 0 |
| sub-35 | 0 | 0 | 0 | 0 |
| sub-36 | 0 | 0 | 0 | 0 |
| sub-37 | 1 | 0 | 0 | 1 |
| sub-38 | 0 | 0 | 0 | 0 |
| sub-39 | 0 | 0 | 0 | 0 |
| sub-40 | 0 | 0 | 0 | 0 |
| sub-41 | 0 | 0 | 0 | 0 |
| sub-42 | 0 | 0 | 0 | 0 |
| sub-43 | 0 | 1 | 0 | 1 |
| sub-44 | 0 | 0 | 0 | 0 |
| sub-45 | 0 | 0 | 0 | 0 |
| sub-46 | 0 | 0 | 1 | 1 |
| sub-47 | 0 | 1 | 0 | 1 |
| sub-48 | 1 | 0 | 0 | 1 |
| sub-49 | 0 | 0 | 0 | 0 |
| sub-50 | 0 | 0 | 0 | 0 |
| sub-51 | 0 | 0 | 0 | 0 |
| sub-52 | 0 | 0 | 0 | 0 |
| sub-53 | 0 | 0 | 0 | 0 |
| sub-54 | 0 | 0 | 0 | 0 |
| sub-55 | 0 | 0 | 0 | 0 |
| sub-56 | 1 | 0 | 1 | 1 |
| sub-57 | 0 | 0 | 0 | 0 |
| sub-58 | 0 | 0 | 0 | 0 |
| sub-59 | 0 | 0 | 0 | 0 |
| sub-60 | 0 | 0 | 0 | 0 |

Score of 0: “Low frequency” defined as “no use”, “less than monthly use” or “one to three times a month”, in the last 12 months, corresponding to scores of 0-2 in frequency dimension on the Diagnostic Interview for Psychoses.

Score of 1: “High Frequency” defined as use of substance “greater than 1-2 times per week” or “daily use” for past 12 months, corresponding to score of 3-4 in frequency dimension on the Diagnostic Interview for Psychoses.

Participants were given a score of 1 on substance use if they had used any substance at high frequency.

Sub-01 to Sub-30: Early Psychosis

Sub-31 to Sub-59: Healthy Control

Sub-60: Healthy control excluded from analysis due to motion artefact

**Supplementary References**

1 Bush, G. & Shin, L. M. The Multi-Source Interference Task: an fMRI task that reliably activates the cingulo-frontal-parietal cognitive/attention network. *Nature protocols* **1**, 308-313 (2006).

2 Eriksen, B. A. & Eriksen, C. W. Effects of noise letters upon the identification of a target letter in a nonsearch task. *Perception & psychophysics* **16**, 143-149 (1974).

3 Esteban, O. *et al.* fMRIPrep: a robust preprocessing pipeline for functional MRI. *Nature methods* **16**, 111-116 (2019).

4 Gorgolewski, K. *et al.* Nipype: a flexible, lightweight and extensible neuroimaging data processing framework in python. *Frontiers in neuroinformatics* **5**, 13 (2011).

5 Tustison, N. J. *et al.* N4ITK: improved N3 bias correction. *IEEE transactions on medical imaging* **29**, 1310-1320 (2010).

6 Avants, B., Anderson, C., Grossman, M. & Gee, J. Symmetric normalization for patient-specific tracking of longitudinal change in frontotemporal dementia. *Med Image Anal* **12**, 26-41 (2008).

7 Zhang, Y., Brady, M. & Smith, S. Segmentation of brain MR images through a hidden Markov random field model and the expectation-maximization algorithm. *IEEE transactions on medical imaging* **20**, 45-57 (2001).

8 Fischl, B., Sereno, M. I. & Dale, A. M. Cortical surface-based analysis: II: inflation, flattening, and a surface-based coordinate system. *Neuroimage* **9**, 195-207 (1999).

9 Klein, A. *et al.* Mindboggling morphometry of human brains. *PLoS computational biology* **13**, e1005350 (2017).

10 Fonov, V., Evans, A., Mckinstry, R., Almli, C. & Collins, D. (2009).

11 Cox, R. W. AFNI: software for analysis and visualization of functional magnetic resonance neuroimages. *Computers and Biomedical research* **29**, 162-173 (1996).

12 Greve, D. N. & Fischl, B. Accurate and robust brain image alignment using boundary-based registration. *Neuroimage* **48**, 63-72 (2009).

13 Jenkinson, M. Bannister P, Brady M, and Smith S. *Improved optimization for the robust and accurate linear registration and motion correction of brain images. Neuroimage* **17**, 825-841 (2002).

14 Power, J. D. *et al.* Methods to detect, characterize, and remove motion artifact in resting state fMRI. *Neuroimage* **84**, 320-341 (2014).

15 Behzadi, Y., Restom, K., Liau, J. & Liu, T. T. A component based noise correction method (CompCor) for BOLD and perfusion based fMRI. *Neuroimage* **37**, 90-101 (2007).

16 Satterthwaite, T. D. *et al.* An improved framework for confound regression and filtering for control of motion artifact in the preprocessing of resting-state functional connectivity data. *Neuroimage* **64**, 240-256 (2013).

17 Lanczos, C. A precision approximation of the gamma function. *Journal of the Society for Industrial and Applied Mathematics, Series B: Numerical Analysis* **1**, 86-96 (1964).

18 Abraham, A. *et al.* Machine learning for neuroimaging with scikit-learn. *Frontiers in neuroinformatics* **8**, 14 (2014).

19 Avants, B. B., Tustison, N. & Song, G. Advanced normalization tools (ANTS). *Insight j* **2**, 1-35 (2009).

20 Schaefer, A. *et al.* Local-global parcellation of the human cerebral cortex from intrinsic functional connectivity MRI. *Cerebral cortex* **28**, 3095-3114 (2018).

21 Tournier, Calamante, F. & Connelly, A. in *Proceedings of the international society for magnetic resonance in medicine.* (Ismrm).

22 Smith, R. E., Tournier, J.-D., Calamante, F. & Connelly, A. SIFT2: Enabling dense quantitative assessment of brain white matter connectivity using streamlines tractography. *Neuroimage* **119**, 338-351 (2015).

23 Roberts, J. A., Perry, A., Roberts, G., Mitchell, P. B. & Breakspear, M. Consistency-based thresholding of the human connectome. *NeuroImage* **145**, 118-129 (2017).

24 Seguin, C., Van Den Heuvel, M. P. & Zalesky, A. Navigation of brain networks. *Proceedings of the National Academy of Sciences* **115**, 6297-6302 (2018).

25 Rubinov, M. & Sporns, O. Complex network measures of brain connectivity: uses and interpretations. *Neuroimage* **52**, 1059-1069 (2010).

26 Tian, Y., Zalesky, A., Bousman, C., Everall, I. & Pantelis, C. Insula Functional Connectivity in Schizophrenia: Subregions, Gradients, and Symptoms. *Biological psychiatry. Cognitive neuroscience and neuroimaging* **4**, 399-408, doi:10.1016/j.bpsc.2018.12.003 (2019).

27 Shine, J. M. *et al.* Computational models link cellular mechanisms of neuromodulation to large-scale neural dynamics. *Nature Neuroscience*, doi:10.1038/s41593-021-00824-6 (2021).

28 Organization, W. H. *The ICD-10 classification of mental and behavioural disorders: clinical descriptions and diagnostic guidelines*. (World Health Organization, 1992).
